# Common Immunosuppressive Monotherapy for Graves’ Ophthalmopathy: A Meta-Analysis

**DOI:** 10.1371/journal.pone.0139544

**Published:** 2015-10-15

**Authors:** Pei Mou, Li-Hong Jiang, Yun Zhang, Yu-Zhen Li, Heng Lou, Cheng-Cheng Zeng, Qiu-Hong Wang, Jin-Wei Cheng, Rui-Li Wei

**Affiliations:** 1 Department of Ophthalmology, Shanghai Changzheng Hospital, Second Military Medical University, Shanghai, China; 2 Department of Ophthalmology, Shanghai Zhabei District Central Hospital, Shanghai, China; Uppsala University, SWEDEN

## Abstract

**Background:**

Several immunosuppressive therapeutic regimens are widely used to treat Graves’ ophthalmopathy (GO), including oral glucocorticoids (OGC), intravenous glucocorticoids (IVGC), retrobulbar injections of glucocorticoids (ROGC) and orbital radiotherapy (OR). The priority among these is unknown. This meta-analysis investigated the efficacy and tolerability of the above regimens.

**Methods:**

The PubMed, EMBASE, and Cochrane Library databases and the Chinese Biomedicine Database were searched up to November 18, 2014. Randomized controlled trials (RCTs) comparing monotherapies (OGC, IVGC, ROGC and OR) in patients with moderate-to-severe active GO were selected. The main efficacy measures were the response rate, the standard mean difference (SMD) in the reduction in the clinical activity score (CAS) and the mean difference (MD) in proptosis from baseline to the end of treatment. The main tolerability measure was the risk ratio (RR) for adverse events. The pooled estimates and 95% confidence intervals (95% CIs) were calculated using the RevMan software, version 5.1.

**Results:**

Seven published RCTs involving 328 participants were included in the present meta-analysis, including IVGC versus OGC (3 trials), ROGC versus OGC (3 trials) and OR versus OGC (1 trial). IVGC was more effective than OGC in response rate (RR = 1.48, 95% CI = 1.18–1.87) and had an obvious CAS reduction (SMD = 0.69, 95% CI = 0.13–1.25). IVGC caused fewer adverse events than OGC. ROGC and OGC had no statistically significant difference in response rate (RR = 1.16, 95% CI = 0.94–1.42). OR also did not differ significantly compared with OGC (RR = 0.93, 95% CI = 0.54–1.60). ROGC and OR had fewer adverse events, such as weight gain, compared with OGC.

**Conclusions:**

For patients with GO in the moderate-to-severe active phase, current evidence gave priority to IVGC, which had a statistically significant advantage over OGC and caused fewer adverse events. ROGC and OR did not provide greater efficacy than OGC, although better tolerability and fewer adverse events were shown.

## Introduction

Graves’ ophthalmopathy, also called thyroid-associated ophthalmopathy, is an organ specific autoimmune disease representing the most common extrathyroidal manifestation of hyperthyroidism. It may also occur in patients with hypothyroidism or euthyroidism[1–4]. The morbidity rate for GO is 19:100,000 per year[5]. It can lead to ocular signs and symptoms including eyelid retraction, lid lag, gritty eye, proptosis, motility restriction, exposure keratopathy and even vision loss. These phenomena decrease the patient quality of life to different extents[6–8].

GO is a large challenge. Its pathogenesis is not well understood, and its management remains controversial[3]. The European Group on Graves’ Orbitopathy (EUGOGO) came to the consensus that all patients with GO, except for the mildest cases, should be referred to multidisciplinary clinics for further assessment and management. If the ophthalmopathy is active, the treatment of choice is intravenous glucocorticoids with or without OR. If in the stable phase or in an emergency (sight-threatening or corneal breakdown), surgical decompression is considered[9].

In the past six decades, oral glucocorticoids have been the most common and widely used immunosuppressants to treat active and moderate-to-severe GO[10–12]. In addition, the use of glucocorticoids given intravenously (i.v.) or injected locally has been widespread. Glucocorticoids play a positive role in reducing the inflammation and congestion of the orbital tissue, thus attempting to prevent the progression of the autoimmune disease. However, they have several side effects due to the amount and duration of drug treatment, including liver dysfunction, weight gain, and cushingoid features [13,14]. For more than 90 years, orbital radiotherapy was also commonly used to treat GO[15]. The most effective and best-tolerated monotherapy for patients with GO is unknown. Therefore, we performed a quantitative review of the evidence and present the following meta-analysis.

## Methods

All randomized controlled trials (RCTs) assessing the efficacy and tolerability of single therapeutic regimens were identified, reviewed and included. Widely accepted methodological recommendations were followed in the present meta-analysis, which was performed according to a predetermined protocol[16–18] using standard systematic review techniques as outlined in the Cochrane Handbook for Systematic Reviews of Interventions and the PRISMA Statement[19,20].

### Outcome Measures

The main outcome measure was the response rate (i.e., the ratio of responders to the total number of participants). When the response rate was reported, we used it directly. If not available, the relevant improvement in clinical parameters was identified as the response to therapy (e.g., a decrease in proptosis and an eyelid retraction of 2 mm or greater, an improvement in the grade of orbital soft tissue swelling, the disappearance of diplopia in the primary gaze, and/or the improvement of eye movement and visual acuity). The secondary outcome measure was the reduction in the clinical activity score from the baseline to the end of follow-up. A reduction in proptosis was also considered.

Tolerability was assessed by calculating the proportion of patients experiencing adverse events in each regimen, including cushingoid features, weight gain, hypertension, gastritis, hyperglycemia and palpitation. Extremely adverse events were particularly recorded.

### Search Strategy

All RCTs were identified through a systematic search consisting of (1) an electronic search of PubMed, EMBASE, the Cochrane library and the Chinese Biomedicine Database and (2) manual searches of the reference lists of original reports and review articles that were retrieved via the electronic searches. A broad search strategy combined terms related to Graves’ ophthalmopathy (including a MeSH search using the exploded term ‘Graves’ ophthalmopathy’ and a keyword search using the words ‘thyroid associated ophthalmopathy’ and ‘thyroid eye disease’), terms related to glucocorticoids (including a MeSH search using the exploded term ‘glucocorticoids’ and a keyword search using the words ‘methylprednisolone’ and ‘prednisone’), and terms related to orbital radiotherapy (including a MeSH search using the exploded term ‘radiotherapy’ and a keyword search using ‘orbital radiotherapy’). The search was limited to clinical trials. Google Scholar was also used to obtain information. This study was performed in accordance with the PRISMA statement checklist (**S1 Text**).

### Trial Selection

Published clinical trials were extracted based on the following protocol-determined selection criteria: (1) study design: randomized, controlled clinical trials; (2) population: patients diagnosed with active and moderate-to-severe GO; (3) intervention: two of the following were included and used separately: IVGC, OGC, ROGC and OR; (4) outcome variables: at least two of the outcome variables, including response rate, reduction in the CAS and/or proptosis, were monitored from the baseline to the end of follow-up.

After the primary searches, three review authors (P.M., L.H.J., Y.Z.) worked independently to assess whether the articles were eligible based on their titles and abstracts. Then, we obtained the potentially relevant manuscripts and assessed each independently according to the definitions in the criteria. Only trials meeting the above-mentioned criteria were assessed for methodological quality. To avoid duplicate publications, we only included the most recent series in the case of data collection from the same study population.

### Data Extraction

Data were extracted according to the customized protocol by three independent authors (P.M., L.H.J., Y.Z.). Any disagreement was resolved by discussion. We used a customized form for data extraction. The following data were recorded and extracted: authors, the time of publication, information on study design (randomization, allocation concealment, intention-to-treat analysis, double or single blind), trial location, follow-up time, severity, CAS, patient age, sex, race, all outcome measures and other essential information. In addition, we noted the proportion of withdrawals and the number of patients undergoing adverse events.

### Qualitative Assessment

Three authors (in duplicate by P.M., L.H.J., Y.Z.) used standard criteria (allocation concealment, blinding, intention to treat analysis, withdrawals) to appraise trial quality in addition to quantitative quality assessment using the scoring system proposed by Jadad[21]. The quality scoring system was as follows: (1) allocation concealment, coded as adequate (1 score) and inadequate or unclear (0 score); (2) blinding, coded as double-blind (2 scores), single-blind (1 score), and open label (0 score); (3) intention to treat analysis, coded as used (1 score) and not used or unable to assess (0 score); (4) withdrawal or loss to follow-up, coded as given (1 score) and not given (0 score).

### Statistical Analysis

**Fig 1:**
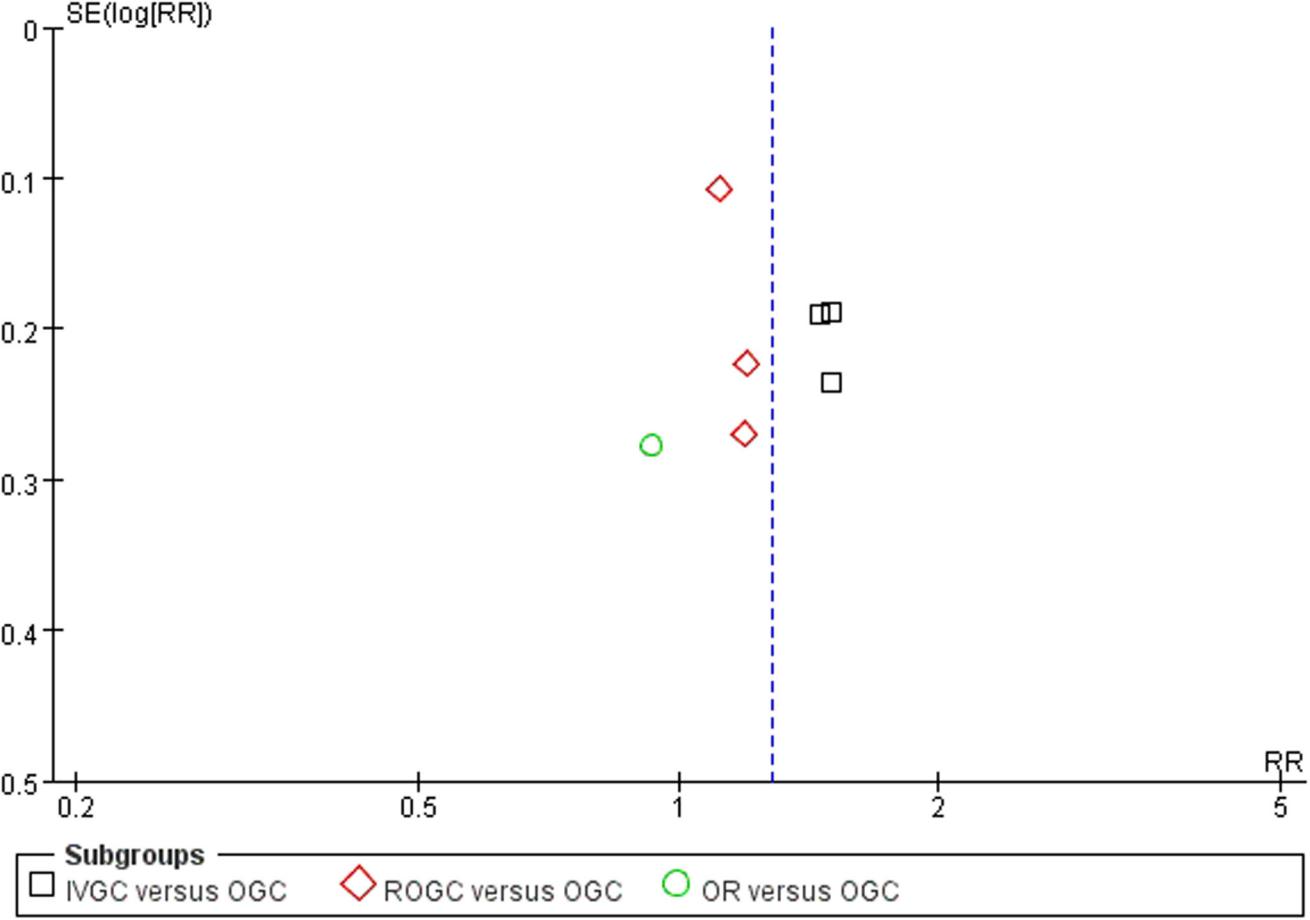
Funnel plot of the response rate in the present meta-analysis. SE = standard error; RR = risk ratio; IVGC = intravenous glucocorticoids; OGC = oral glucocorticoids; ROGC = retrobulbar injections of glucocorticoids; OR = orbital radiotherapy.

The statistical analysis was performed using the RevMan software, version 5.1 (the Cochrane Collaboration, Oxford, UK). Data were assessed based on an intent-to-treat (ITT) principle. The standard mean difference was estimated for continuous outcomes, and the risk ratio was calculated for dichotomous outcomes. All were demonstrated using the 95% confidence intervals. A Q value < 0.05 was considered statistically significant. We reported the heterogeneity across eligible studies checked by the Q-value. We also reported the I^2^ metric measure of inconsistency to assess the heterogeneity regardless of the number of studies[22]. If P > 0.1 (I^2^ < 50%), no heterogeneity was detected. In this case, we used the Mantel-Haenszel fixed effects model to assess the combined results in a meta-analysis[23]. Otherwise, the reasons for the existing heterogeneity were searched and checked. No publication bias existed as demonstrated by the funnel plot [24] (**Fig 1**).

## Results

**Fig 2:**
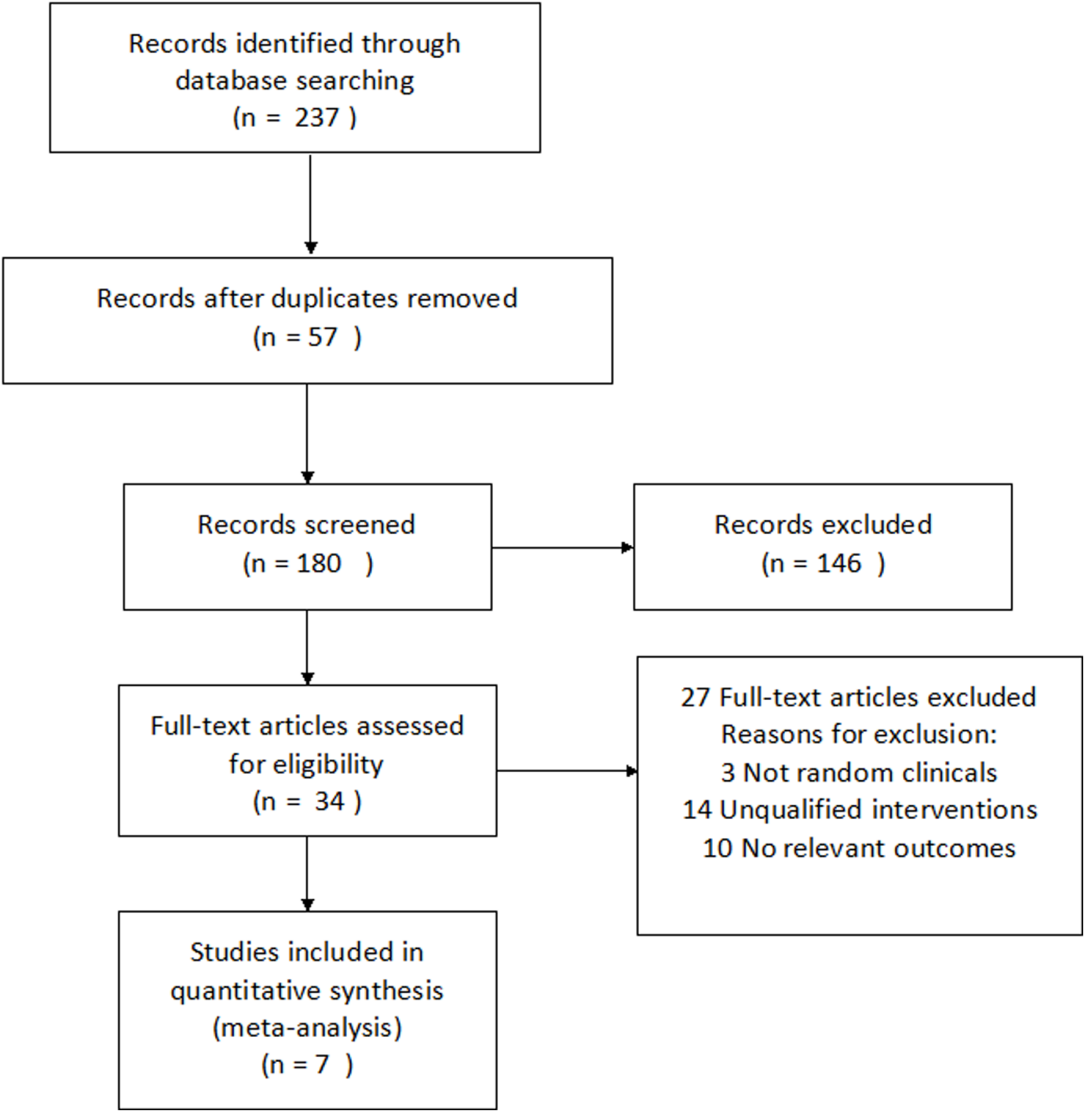
The selection flowchart of the studies included in the present meta-analysis.

Seven RCTs involving 328 participants fulfilled our inclusion criteria[25–31]. The flow of the study selection in our meta-analysis is summarized in **Fig 2**.

Information demonstrating baseline characteristics is shown in **Table 1**. All seven trials were prospective and parallel. Four were open label, two were single blind and one was double blind. Seven trials were carried out in single centers located in Turkey (1), Germany (1), Italy (1), Egypt (1), China (2) and Holland (1). The follow-up time of the studies ranged from 3 months to 12 months. The mean age of the patients from the seven separate studies ranged from 34 to 50. Among the studies, except for Liu 2006 (in which sex was not mentioned), there were 87 males and 206 females.

**Table 1:** Baseline characteristics of randomized clinical trials included in the present meta-analysis.

Trials	Design Center Location	Treatment NO	Control NO	Follow-up time (months)	Mean Age	Sex (M/F)	Severity	CAS		
								7	10	other
**Aktaran 2006**	SB-P Single Turkey	IVGC 25	OGC 27	3	43	24/28	moderate-to-severe		5.2/5	
**Kalaly 2005**	SB-P Single Germany	IVGC 35	OGC 35	6	50	21/49	moderate-to-severe	5		
**Macchia 2001**	OL-P Single Italy	IVGC 25	OGC 26	12	44	11/40	NA	4.43/ 2.65		
**Alkawas 2010**	OL-P Single Egypt	ROGC 12	OGC 12	6	34	8/16	moderate-to-severe	5/4.75		
**Gui-qin Liu 2006**	OL-P Single China	ROGC 15	OGC 15	6	35.8	UA	moderate			Sum of ranks (CAS): 27.56/26.58
**Jing-ming Zhang 2005**	OL-P Single China	ROGC 20	OGC 20	6	39.28	14/26	moderate-to-severe			NA
**Prummel 1993**	DB-P Single Holland	OR 28	OGC 28	6	47	9/47	moderate-to-severe	5.2		

NO = number of patients; SB-P = single-blind parallel; DB-P = double-blind parallel; OL-P = open label parallel; NA = unable to assess; IVGC = intravenous glucocorticoids; OGC = oral glucocorticoids; ROGC = retrobulbar injection of glucocorticoids; OR = orbital radiotherapy; M = male; F = female; CAS = clinical activity score.

The seven included studies had a mean score of 3.14 using the scoring system developed by Jadad. One trial scored 5, three scored 4, two scored 2, and one scored 1. Allocation concealment was adequate in four trials and unclear in the other three. Participants were blinded in only one trial, investigators were blinded in two and examiners in three. Proportions of withdrawal ranged from 0% to 17%. The ITT principle was used to analyze patients in six studies. These are demonstrated in **Table 2**.

**Table 2:** Methodological quality of randomized clinical trials included in the present meta-analysis.

	Allocation		Blinded				Quality
Trials	concealment	Participants	Investigators	Examiners	ITT	Withdrawal	score
**Aktaran 2006**	Adequate	No	No	Yes	Yes	0%	4
**Kalaly 2005**	Adequate	No	No	Yes	Yes	0%	4
**Macchia 2001**	Unclear	No	No	No	NA	6%	1
**Alkawas 2010**	Adequate	No	Yes	Yes	Yes	17%	4
**Gui-qin Liu 2006**	Unclear	No	No	No	Yes	0%	2
**Jing-ming Zhang 2005**	Unclear	No	No	No	Yes	0%	2
**Prummel 1993**	Adequate	Yes	Yes	No	Yes	0%	5

ITT = intention to treat analysis; NA = unable to assess.

## Efficacy

### Response rate

**Fig 3:**
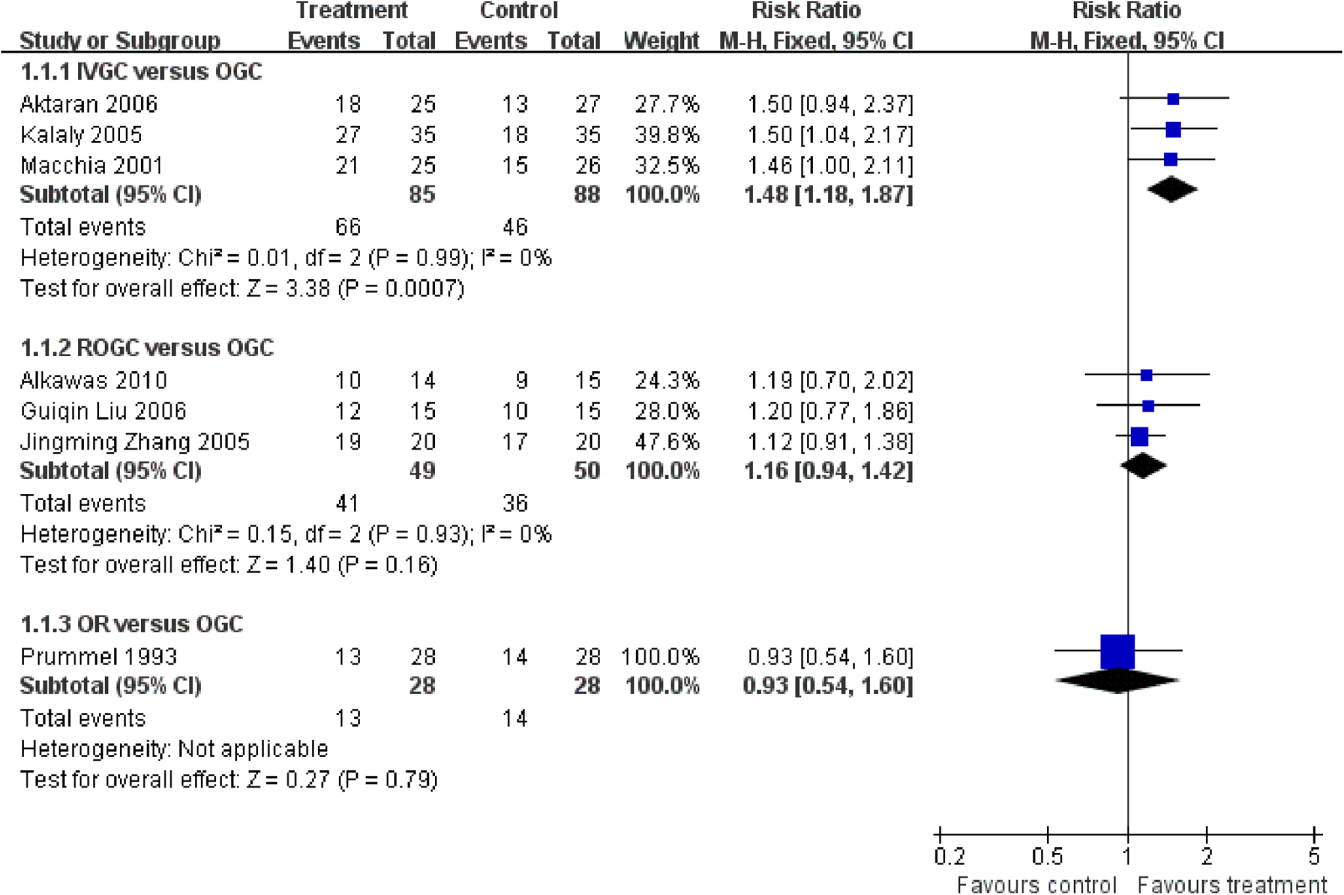
Forest plot of the response rate at the end of follow-up. IVGC = intravenous glucocorticoids; OGC = oral glucocorticoids; ROGC = retrobulbar injections of glucocorticoids; OR = orbital radiotherapy.

All seven included studies reported the response rate (**Fig 3**).

Three studies compared IVGC with OGC, including Aktaran 2006 (25:27), Kalaly 2005 (35:35) and Macchia 2001 (25:26). No heterogeneity was found (Q = 0.01, P = 0.99, I^2^ = 0%). The numbers of events versus total numbers in IVGC and OGC were 66:85 and 46:88, respectively. In the IVGC group, the response rate was significantly higher than in the OGC group (RR = 1.48, 95% CI = 1.18–1.87, P = 0.0007). In the OGC group in Macchia 2001, three patients had to withdraw from the treatment due to severe signs or symptoms of hypercortisolism, including hyperglycemia, polymenorrhea and central obesity.

Treatment with ROGC showed a better response rate than OGC based on the data from three trials, including Alkawas 2010 (14:15), Guiqin Liu 2006 (15:15), and Jingming Zhang 2005 (20:20). No heterogeneity was found (Q = 0.15, P = 0.93, I^2^ = 0%). The number of events versus total numbers in ROGC and OGC were 41:49 and 36:50, respectively. No statistically significant difference was demonstrated between the two groups (RR = 1.16, 95% CI = 0.94–1.42, P = 0.16). In the Alkawas 2010 study, 29 patients suffering from GO were included and randomized into the ROGC group (14) or OGC group (15). Only 12 patients in each group completed the study, but the reasons were not mentioned.

Only one trial (Prummel 1993) compared OR versus OGC; thus, heterogeneity was not applicable. The numbers of events versus total numbers in these groups were 13:28 and 14:28, respectively. No statistical significance was found, with a RR of 0.93 and 95% CI of 0.54 to 1.60. All patients completed the study.

### Reduction of CAS

**Fig 4:**
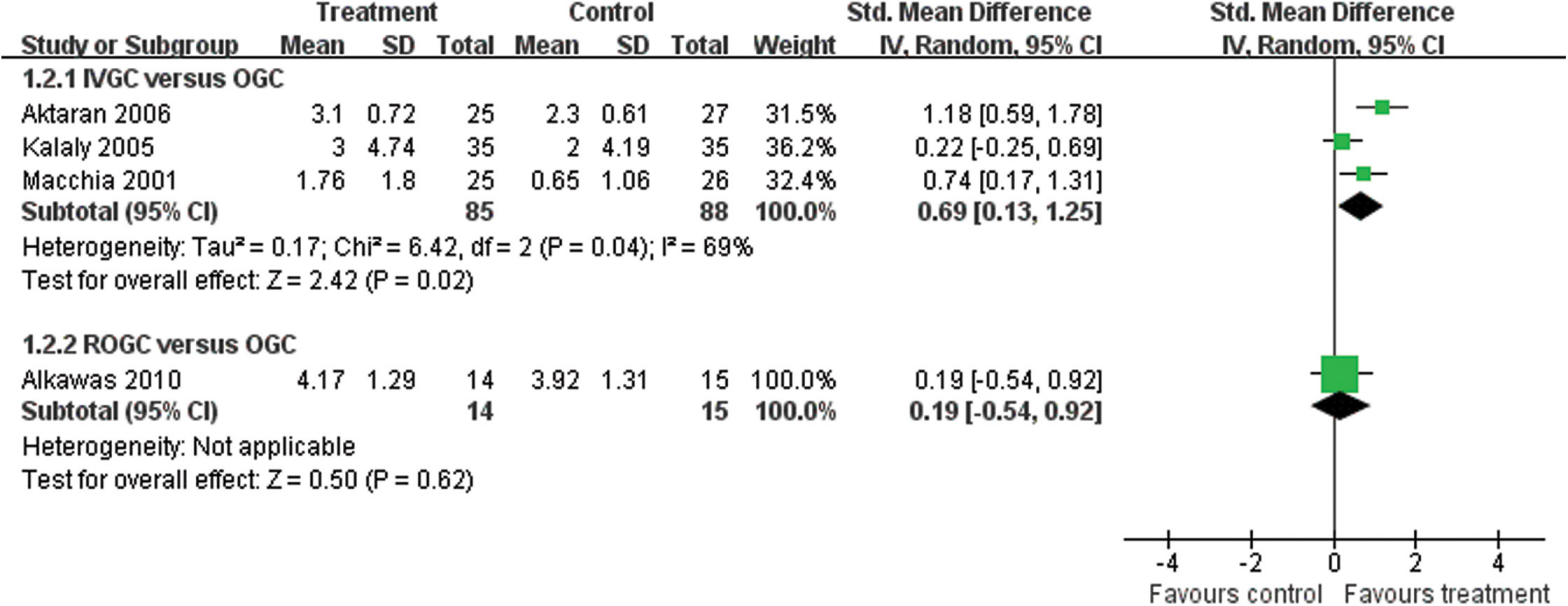
Forest plot of the reduction of CAS at the end of follow-up. SD = standard deviation; IVGC = intravenous glucocorticoids; OGC = oral glucocorticoids; ROGC = retrobulbar injections of glucocorticoids.

Four studies mentioned the reduction of CAS from baseline to the end of the follow-up, including IVGC versus OGC (Aktaran 2006, Kalaly 2005, Macchia 2001) and ROGC versus OGC (Alkawas 2010). Details are shown in **Fig 4.**

The heterogeneity between the IVGC group and the OGC group was higher than 50% (Q = 6.42, P = 0.04, I^2^ = 69%). Thus, we used a random model to assess the reduction of CAS to obtain more appropriate results. IVGC offered a stronger power in reducing CAS than OGC (SMD = 0.69; 95%CI = 0.13–1.25; P = 0.02). We found that the standard difference in Kalaly 2005 (4.74 and 4.19 in each group) was much larger than the others, which may be due to the higher initial dose of oral prednisolone than in the other two studies. If we excluded this study, the heterogeneity changed noticeably (Q = 1.11, P = 0.29, I^2^ = 10%). The advantage of IVGC was also elevated (SMD = 0.96, 95% CI = 0.52–1.39, P < 0.0001).

Alkawas 2010 compared ROGC with OGC (14:15). Heterogeneity was not applicable, with an SMD of 0.19 and 95% CI of -0.54 to 0.92. No statistically significant difference existed between the two groups.

### Reduction of proptosis

**Fig 5:**
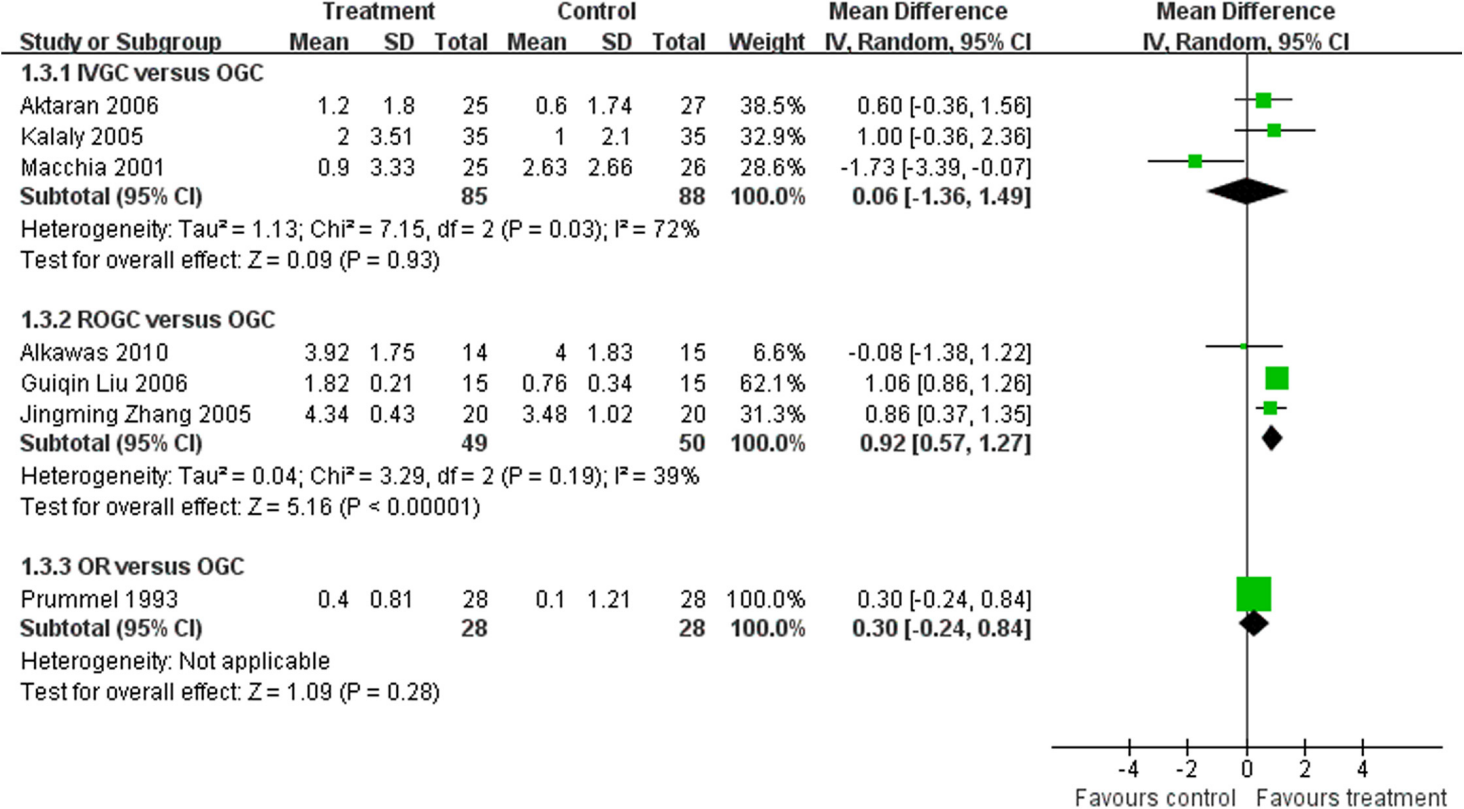
Forest plot of the reduction of proptosis at the end of follow-up. SD = standard deviation; IVGC = intravenous glucocorticoids; OGC = oral glucocorticoids; ROGC = retrobulbar injections of glucocorticoids; OR = orbital radiotherapy.

All seven studies described the reduction of proptosis. A random model was used because of the high heterogeneity between IVGC and OGC (Q = 7.11, P = 0.03, I^2^ = 72%) (**Fig 5**).

Three studies compared the reduction of proptosis between IVGC and OGC (Aktaran 2006, Kalaly 2005, Macchia 2001). The high heterogeneity could be reduced to 0% if we excluded Macchia 2001, which may be due to the higher single dose and cumulative dose of i.v. methylprednisolone for GO patients and the longer follow-up time, which were different from the other two studies. Taking Aktaran 2006 and Kalaly 2005 into account, the MD was 0.73 and the 95% CI was -0.05 to 1.52, meaning no significant difference existed.

All three studies of the ROGC versus OGC comparison mentioned the reduction of proptosis. No heterogeneity existed (Q = 3.29, P = 0.19, I^2^ = 39%). ROGC had a significant advantage over OGC (MD = 0.92, 95% CI = 0.57–1.27).

Prummel 1993 also reported this as the secondary outcome measure of the therapy. OR and OGC showed no statistically significant difference, although accurate measurements had be performed (MD = 0.30, 95% CI = -0.24–0.84).

### Tolerability

We summarized the common side effects and adverse events mentioned in the seven included studies. In each comparison, a random or fixed model was used depending on the heterogeneity across the studies **(Table 3).**

**Table 3:** Overall effect of adverse events in the present meta-analysis.

Adverse events	NO	Crude rate, n/N		RR (95% CI)	Heterogeneity	RR (95% CI)
		IVGC	OGC			
**Weight gain**	3	3/85	15/88	0.24 (0.07, 0.79)	P = 0.35	P = 0.02
**Cushingoid features**	2	1/50	8/53	0.19 (0.03, 1.01)	P = 0.29	P = 0.05
**Palpitation**	2	7/60	1/62	5.15 (0.93, 28.57)	P = 0.73	P = 0.06
**Hypertension**	3	0/85	11/85	0.12 (0.02, 0.66)	P = 0.82	P = 0.01
**Gastritis**	3	5/85	8/88	0.67 (0.24, 1.89)	P = 0.15	P = 0.45
**Hyperglycemia**	2	3/50	5/53	0.69 (0.02, 19.41)	P = 0.06	P = 0.83
		ROGC	OGC			
**Weight gain**	1	0/14	8/15	0.06 (0.00, 1.00)	NA	P = 0.05
**Hypertension**	1	1/14	6/15	0.18 (0.02, 1.30)	NA	P = 0.09
**Gastritis**	1	1/14	9/15	0.12 (0.02, 0.82)	NA	P = 0.03
**Hyperglycemia**	1	0/14	4/15	0.12 (0.01, 2.02)	NA	P = 0.14
		OR	OGC			
**Weight gain**	1	3/28	12/28	0.25 (0.08, 0.79)	NA	P = 0.02
**Cushingoid features**	1	0/28	14/28	0.03 (0.00, 0.55)	NA	P = 0.02
**Palpitation**	1	2/28	5/28	0.40 (0.08, 1.89)	NA	P = 0.25
**Hypertension**	1	0/28	2/28	0.20 (0.01, 3.99)	NA	P = 0.29
**Gastritis**	1	2/28	5/28	0.40 (0.08, 1.89)	NA	P = 0.25

NO = number of studies; n = number of patients with adverse events; N = number of patients; RR = risk ratio; 95% CI = 95% confidence interval; NA = not applicable.

Five studies covered the weight gain ratios, including IVGC versus OGC (Aktaran 2006, Kalaly 2005, Macchia 2001), ROGC versus OGC (Alkawas 2010) and OR versus OGC (Prummel 1993). IVGC, ROGC and OR all showed a statistically significant lower rate of weight gain occurrence than OGC, with RRs of 0.24 (95% CI = 0.07–0.79, P = 0.02), 0.06 (95% CI = 0.00–0.92, P = 0.04) and 0.25 (95% CI = 0.08–0.79, P = 0.02), respectively.

Cushingoid features were mentioned in three trials, including IVGC versus OGC (Alkaran 2006, Macchia 2001) and OR versus OGC (Prummel 1993). No statistically significant difference existed between IVGC and OGC (RR = 0.19, 95% CI = 0.03–1.01, P = 0.05). However, OR was better than OGC in terms of cushingoid features (RR = 0.03, 95% CI = 0.00–0.55, P = 0.02).

Five studies reported the hypertension incidence rate, including IVGC versus OGC (Aktaran 2006, Kalaly 2005, Macchia 2001), ROGC versus OGC (Alkawas 2010) and OR versus OGC (Prummel 1993). In patients receiving IVGC, the incidence rate of hypertension was statistically lower than in patients receiving OGC (RR = 0.12, 95% CI = 0.02–0.66, P = 0.01). No statistically significant difference was found in the other two compared groups, with RRs of 0.18 (95% CI = 0.02–1.30) and 0.20 (95% CI = 0.01–3.99), respectively.

Gastritis was mentioned in five trials, including IVGC versus OGC (Alkaran 2006, Kalaly 2005, Macchia 2001), ROGC versus OGC (Alkawas 2010) and OR versus OGC (Prummel 1993). Gastritis was statistically rarer in ROGC than in OGC (RR = 0.11, 95% CI = 0.02–0.75, P = 0.02). No statistically significant difference existed within the other two compared groups.

## Discussion

In this meta-analysis, we reviewed seven RCTs comparing IVGC, ROGC, OGC and OR as monotherapies for patients with active and moderate-to-severe GO.

Responses were observed in both the IVGC and OGC groups, with rates of 66/85 and 46/88, respectively. However, patients receiving IVGC therapy had a statistically better response rate than those treated with OGC, which may be due to the higher single treatment and cumulative dose of glucocorticoids. Moreover, glucocorticoids may achieve a more rapid immune suppression effect through IV drip. IVGC showed stronger efficacy than OGC in controlling acute inflammation, whose signs and symptoms were mentioned in the CAS scoring system. There was no significant difference in the reduction of proptosis in our meta-analysis. More studies are needed to verify this conclusion. In addition, IVGC demonstrated its advantage over OGC when focusing on hypertension and weight gain, which may be associated with the route and interval pulse dosing method.

Data extracted from the included trials revealed that ROGC and OGC were both effective, with rates of 41/49 and 36/50, respectively. No statistically significant difference was found when comparing the efficacy of these two regimens. ROGC was better in proptosis reduction, but no statistically significant difference existed in CAS reduction. Incidence rates of gastritis and weight gain were statistically lower in the ROGC group compared with the OGC group. These findings proved that ROGC can improve the symptoms of inflammation as well as OGC without the unacceptable rates of local complications. Systemic side effects also decreased.

Recent years have witnessed important discussions on the role of OR, particularly questioning its efficacy and safety. In our meta-analysis, OR and OGC had no significantly different effects on response rate and proptosis reduction, but data summing up the decreased CAS were wanting. OR caused fewer Cushingoid features and less weight gain than OGC.

These results could be supported in previous meta-analyses. One meta-analysis presented by Gao[32] included eight studies involving 376 patients comparing the efficacy of IVGC and OGC with or without assist therapies (OR or surgical decompression). Gao demonstrated the priority of IVGC, as treated as the mainstay and first-line treatment for GO. Taking the potential adverse events into consideration, careful selection of the patients before treatment and limitation of total cumulative dose of glucocorticoids are essential[14,33]. Shachaf Shiber[34] concluded that a standard dose of prednisone (0.4–0.5 mg/kg tapered over three months) was the best validated regimen, which should be used in patients with mild-to-moderate GO who had high risk of progression. Low-dose prednisone (0.2–0.3 mg/kg tapered over 4–5 weeks) can be used in patients with mild GO and in patients without preexisting GO who had risk factors and were selected for GC prophylaxis. In Ebner’s study[35], ROGC was effective with better area of binocular vision without diplopia. Extraocular muscles were size-reduced compared with the control group, which received no treatment. Bordaberry[36] treated GO patients in the moderate-to-severe stage with associated optic neuropathy. Bordaberry received a satisfactory result in that 66% of participants improved with ROGC therapy. Rajendram R[37] summarized the efficacy of OR for GO patients and found no difference between OR and steroid monotherapy in any single trial. When comparing the outcomes of disease severity such as total eye score, NOSPECS score and ophthalmopathy index, better outcomes have been observed with combinations of OR and steroids versus steroids alone. Moreover, adverse events caused by OR were local and mild, supporting the safety and tolerability of OR, although long-term data were needed. Viani’s data[38] showed that OR should be offered as a valid therapeutic option to patients suffering moderate-to-severe ophthalmopathy. The effectiveness of OR can be increased by the synergistic interaction with glucocorticoids. Moreover, OR was useful to improve ocular symptoms, excluding intraocular pressure, with no difference in quality of life or cost.

Unlike the previous studies, we compared different monotherapies for GO patients. Not only the response rate but also CAS and proptosis reduction were used as the outcome measures. Tolerability was confirmed through carefully calculating the risk ratio of patients undergoing adverse events.

Credible conclusions were due to the high quality of the evidence. Overall, 328 participants were randomized in the seven studies included in this meta-analysis. Each study was an RCT with level one evidence. Multiple databases and websites were searched to avoid publication bias. We also used funnel plots to detect potential biases. Fortunately, little evidence for such a bias was found.

Although careful work was necessary to obtain accurate results, our present meta-analysis had its own limitations rooted in the designs of the individual trials and the methods used. One limitation was the low number of included trials, especially of OR versus OGC. Papers published in minor languages may have been missed. All these trials were carried out in a single center, and not all were blinded. Some of the trials had unclear allocation concealment. Additionally, one study was scored 1 and two scored 2 on the Jadad scoring system, which were not high enough. The follow-up time of the seven trials ranged from three to 12 months, which were different from each other and were all not long enough to evaluate the long-term effects of treatment.

Thus, high-quality studies carried out in multiple centers with much longer follow-up times and no time or language limits are necessary.

## Conclusion

In conclusion, IVGC remained the first-line treatment for patients with active and moderate-to-severe GO due to its higher response rate and fewer adverse events. ROGC and OR were also recommended for their better tolerability, although the response rates were not increased compared with OGC.

Patients with GO should be referred to multidisciplinary specialist centers to receive comprehensive therapy[9]. Restoring and maintaining thyroid function was the foundation of treatment. Quitting smoking was beneficial in many studies[3,39,40]. Proper therapeutic regimens could be used singly or combined depending on the individual conditions. Quality of life and the mental health of patients also need more concern[3,8].

## Supporting Information

S1 Text PRISMA Checklist. Preferred Reporting Items for Meta-Analyses (PRISMA) statement checklist.
(DOC)

S2 Text Full-text excluded studies. (DOC)

S3 Text Predetermined protocol. (DOC)
