# Supplementary material for: Common Immunosuppressive Monotherapy for Graves’ Ophthalmopathy: A Meta-Analysis
Source: PLoS One. 2015 Oct 15;10(10):e0139544. doi: 10.1371/journal.pone.0139544 (PMC4607493; doi:10.1371/journal.pone.0139544)
Supplement: S2 Text — (DOC) [file pone.0139544.s002.doc]

1 [Graves' ophthalmopathy: natural history and treatment outcomes.](http://www.ncbi.nlm.nih.gov/pubmed/11820071)

[Noth D](http://www.ncbi.nlm.nih.gov/pubmed/?term=Noth D[Author]&cauthor=true&cauthor_uid=11820071)1, [Gebauer M](http://www.ncbi.nlm.nih.gov/pubmed/?term=Gebauer M[Author]&cauthor=true&cauthor_uid=11820071), [Müller B](http://www.ncbi.nlm.nih.gov/pubmed/?term=Müller B[Author]&cauthor=true&cauthor_uid=11820071), [Bürgi U](http://www.ncbi.nlm.nih.gov/pubmed/?term=Bürgi U[Author]&cauthor=true&cauthor_uid=11820071), [Diem P](http://www.ncbi.nlm.nih.gov/pubmed/?term=Diem P[Author]&cauthor=true&cauthor_uid=11820071).

[Swiss Med Wkly.](http://www.ncbi.nlm.nih.gov/pubmed) 2001 Oct 20;131(41-42):603-9.

Not RCT

2 Steroid therapy for Graves' ophthalmopathy

[Hiromatsu Y](http://www.ncbi.nlm.nih.gov/pubmed/?term=Hiromatsu Y[Author]&cauthor=true&cauthor_uid=17154092).

[Nihon Rinsho.](http://www.ncbi.nlm.nih.gov/pubmed/17154092) 2006 Dec;64(12):2279-85.

not RCT

3 Treatment of Graves' Ophthalmopathy with Oral or Intravenous Corticosteroids

[Brauer VF](http://www.ncbi.nlm.nih.gov/pubmed/?term=Brauer VF[Author]&cauthor=true&cauthor_uid=14963657), [Scholz GH](http://www.ncbi.nlm.nih.gov/pubmed/?term=Scholz GH[Author]&cauthor=true&cauthor_uid=14963657).

[Med Klin (Munich).](http://www.ncbi.nlm.nih.gov/pubmed/14963657) 2004 Feb 15;99(2):71-6.

not RCT

4 Treatment of thyroid associated ophthalmopathy with periocular injections of triamcinolone.

Ebner R, Devoto MH, Weil D, Bordaberry M, Mir C, Martinez H, Bonelli L, Niepomniszcze H.

Br J Ophthalmol. 2004 Nov;88(11):1380-6.

Unqualified interventions：blank control

5 Cigarette smoking and treatment outcomes in Graves ophthalmopathy.

[Bartalena L](http://www.ncbi.nlm.nih.gov/pubmed/?term=Bartalena L[Author]&cauthor=true&cauthor_uid=9786811), [Marcocci C](http://www.ncbi.nlm.nih.gov/pubmed/?term=Marcocci C[Author]&cauthor=true&cauthor_uid=9786811), [Tanda ML](http://www.ncbi.nlm.nih.gov/pubmed/?term=Tanda ML[Author]&cauthor=true&cauthor_uid=9786811), [Manetti L](http://www.ncbi.nlm.nih.gov/pubmed/?term=Manetti L[Author]&cauthor=true&cauthor_uid=9786811), [Dell'Unto E](http://www.ncbi.nlm.nih.gov/pubmed/?term=Dell'Unto E[Author]&cauthor=true&cauthor_uid=9786811), [Bartolomei MP](http://www.ncbi.nlm.nih.gov/pubmed/?term=Bartolomei MP[Author]&cauthor=true&cauthor_uid=9786811), [Nardi M](http://www.ncbi.nlm.nih.gov/pubmed/?term=Nardi M[Author]&cauthor=true&cauthor_uid=9786811), [Martino E](http://www.ncbi.nlm.nih.gov/pubmed/?term=Martino E[Author]&cauthor=true&cauthor_uid=9786811), [Pinchera A](http://www.ncbi.nlm.nih.gov/pubmed/?term=Pinchera A[Author]&cauthor=true&cauthor_uid=9786811).

[Ann Intern Med.](http://www.ncbi.nlm.nih.gov/pubmed/9786811) 1998 Oct 15;129(8):632-5.

Unqualified interventions：OR VS OR+OGC

6 High dose intravenous methylprednisolone pulse therapy versus oral prednisone for thyroid-associated ophthalmopathy.

[Kauppinen-Mäkelin R](http://www.ncbi.nlm.nih.gov/pubmed/?term=Kauppinen-Mäkelin R[Author]&cauthor=true&cauthor_uid=12059873), [Karma A](http://www.ncbi.nlm.nih.gov/pubmed/?term=Karma A[Author]&cauthor=true&cauthor_uid=12059873), [Leinonen E](http://www.ncbi.nlm.nih.gov/pubmed/?term=Leinonen E[Author]&cauthor=true&cauthor_uid=12059873), [Löyttyniemi E](http://www.ncbi.nlm.nih.gov/pubmed/?term=Löyttyniemi E[Author]&cauthor=true&cauthor_uid=12059873), [Salonen O](http://www.ncbi.nlm.nih.gov/pubmed/?term=Salonen O[Author]&cauthor=true&cauthor_uid=12059873), [Sane T](http://www.ncbi.nlm.nih.gov/pubmed/?term=Sane T[Author]&cauthor=true&cauthor_uid=12059873), [Setälä K](http://www.ncbi.nlm.nih.gov/pubmed/?term=Setälä K[Author]&cauthor=true&cauthor_uid=12059873), [Viikari J](http://www.ncbi.nlm.nih.gov/pubmed/?term=Viikari J[Author]&cauthor=true&cauthor_uid=12059873), [Heufelder A](http://www.ncbi.nlm.nih.gov/pubmed/?term=Heufelder A[Author]&cauthor=true&cauthor_uid=12059873), [Välimäki M](http://www.ncbi.nlm.nih.gov/pubmed/?term=Välimäki M[Author]&cauthor=true&cauthor_uid=12059873).

[Acta Ophthalmol Scand.](http://www.ncbi.nlm.nih.gov/pubmed/12059873) 2002 Jun;80(3):316-21.

Unqualified interventions：IVGC+OGC vs OGC

7 Orbital radiation therapy for Graves' ophthalmopathy: measuring clinical efficacy and impact.

[Hahn E](http://www.ncbi.nlm.nih.gov/pubmed/?term=Hahn E[Author]&cauthor=true&cauthor_uid=25012831), [Laperriere N](http://www.ncbi.nlm.nih.gov/pubmed/?term=Laperriere N[Author]&cauthor=true&cauthor_uid=25012831), [Millar BA](http://www.ncbi.nlm.nih.gov/pubmed/?term=Millar BA[Author]&cauthor=true&cauthor_uid=25012831), [Oestreicher J](http://www.ncbi.nlm.nih.gov/pubmed/?term=Oestreicher J[Author]&cauthor=true&cauthor_uid=25012831), [McGowan H](http://www.ncbi.nlm.nih.gov/pubmed/?term=McGowan H[Author]&cauthor=true&cauthor_uid=25012831), [Krema H](http://www.ncbi.nlm.nih.gov/pubmed/?term=Krema H[Author]&cauthor=true&cauthor_uid=25012831), [Gill H](http://www.ncbi.nlm.nih.gov/pubmed/?term=Gill H[Author]&cauthor=true&cauthor_uid=25012831), [DeAngelis D](http://www.ncbi.nlm.nih.gov/pubmed/?term=DeAngelis D[Author]&cauthor=true&cauthor_uid=25012831), [Hurwitz J](http://www.ncbi.nlm.nih.gov/pubmed/?term=Hurwitz J[Author]&cauthor=true&cauthor_uid=25012831), [Tucker N](http://www.ncbi.nlm.nih.gov/pubmed/?term=Tucker N[Author]&cauthor=true&cauthor_uid=25012831), [Simpson R](http://www.ncbi.nlm.nih.gov/pubmed/?term=Simpson R[Author]&cauthor=true&cauthor_uid=25012831), [Chung C](http://www.ncbi.nlm.nih.gov/pubmed/?term=Chung C[Author]&cauthor=true&cauthor_uid=25012831)

[Pract Radiat Oncol.](http://www.ncbi.nlm.nih.gov/pubmed/25012831) 2014 Jul-Aug;4(4):233-9. doi: 10.1016/j.prro.2014.02.008. Epub 2014 Apr 3.

Unqualified interventions：

8 A prospective, randomized, double-blind, placebo-controlled study of orbital radiotherapy for Graves' ophthalmopathy.

Gorman CA, Garrity JA, Fatourechi V, Bahn RS, Petersen IA, Stafford SL, Earle JD, Forbes GS, Kline RW, Bergstralh EJ, Offord KP, Rademacher DM, Stanley NM, Bartley GB.

Ophthalmology. 2001 Sep;108(9):1523-34.

Unqualified interventions：OR vs sham OR

9 A randomized controlled trial of orbital radiotherapy versus sham irradiation in patients with mild Graves'ophthalmopathy.

[Prummel MF](http://www.ncbi.nlm.nih.gov/pubmed/?term=Prummel MF[Author]&cauthor=true&cauthor_uid=14715820), [Terwee CB](http://www.ncbi.nlm.nih.gov/pubmed/?term=Terwee CB[Author]&cauthor=true&cauthor_uid=14715820), [Gerding MN](http://www.ncbi.nlm.nih.gov/pubmed/?term=Gerding MN[Author]&cauthor=true&cauthor_uid=14715820), [Baldeschi L](http://www.ncbi.nlm.nih.gov/pubmed/?term=Baldeschi L[Author]&cauthor=true&cauthor_uid=14715820), [Mourits MP](http://www.ncbi.nlm.nih.gov/pubmed/?term=Mourits MP[Author]&cauthor=true&cauthor_uid=14715820), [Blank L](http://www.ncbi.nlm.nih.gov/pubmed/?term=Blank L[Author]&cauthor=true&cauthor_uid=14715820), [Dekker FW](http://www.ncbi.nlm.nih.gov/pubmed/?term=Dekker FW[Author]&cauthor=true&cauthor_uid=14715820), [Wiersinga WM](http://www.ncbi.nlm.nih.gov/pubmed/?term=Wiersinga WM[Author]&cauthor=true&cauthor_uid=14715820).

[J Clin Endocrinol Metab.](http://www.ncbi.nlm.nih.gov/pubmed/14715820) 2004 Jan;89(1):15-20.

Unqualified interventions：OR VS sham OR

10 Effect of steroid pulse therapy with and without orbital radiotherapy on Graves' ophthalmopathy.

[Ohtsuka K](http://www.ncbi.nlm.nih.gov/pubmed/?term=Ohtsuka K[Author]&cauthor=true&cauthor_uid=12614743), [Sato A](http://www.ncbi.nlm.nih.gov/pubmed/?term=Sato A[Author]&cauthor=true&cauthor_uid=12614743), [Kawaguchi S](http://www.ncbi.nlm.nih.gov/pubmed/?term=Kawaguchi S[Author]&cauthor=true&cauthor_uid=12614743), [Hashimoto M](http://www.ncbi.nlm.nih.gov/pubmed/?term=Hashimoto M[Author]&cauthor=true&cauthor_uid=12614743), [Suzuki Y](http://www.ncbi.nlm.nih.gov/pubmed/?term=Suzuki Y[Author]&cauthor=true&cauthor_uid=12614743).

[Am J Ophthalmol.](http://www.ncbi.nlm.nih.gov/pubmed/12614743) 2003 Mar;135(3):285-90.

Unqualified interventions：IVGC+OGC VS OR+OGC

11 Repeated triamcinolone acetonide injection in the treatment of upper-lid retraction in patients with thyroid-associated ophthalmopathy.

[Xu D](http://www.ncbi.nlm.nih.gov/pubmed/?term=Xu D[Author]&cauthor=true&cauthor_uid=22333849), [Liu Y](http://www.ncbi.nlm.nih.gov/pubmed/?term=Liu Y[Author]&cauthor=true&cauthor_uid=22333849), [Xu H](http://www.ncbi.nlm.nih.gov/pubmed/?term=Xu H[Author]&cauthor=true&cauthor_uid=22333849), [Li H](http://www.ncbi.nlm.nih.gov/pubmed/?term=Li H[Author]&cauthor=true&cauthor_uid=22333849).

[Can J Ophthalmol.](http://www.ncbi.nlm.nih.gov/pubmed/22333849) 2012 Feb;47(1):34-41. doi: 10.1016/j.jcjo.2011.12.005.

Reason: no relevant outcomes

12 The blood concentration of intercellular adhesion molecule-1 (sICAM-1) and vascular cell adhesion molecule-1 (sVCAM-1) in patients with active thyroid-associated orbitopathy before and after methylprednisolonetreatment.

[Nowak M](http://www.ncbi.nlm.nih.gov/pubmed/?term=Nowak M[Author]&cauthor=true&cauthor_uid=18205104), [Wielkoszyński T](http://www.ncbi.nlm.nih.gov/pubmed/?term=Wielkoszyński T[Author]&cauthor=true&cauthor_uid=18205104), [Kos-Kudła B](http://www.ncbi.nlm.nih.gov/pubmed/?term=Kos-Kudła B[Author]&cauthor=true&cauthor_uid=18205104), [Marek B](http://www.ncbi.nlm.nih.gov/pubmed/?term=Marek B[Author]&cauthor=true&cauthor_uid=18205104), [Karpe J](http://www.ncbi.nlm.nih.gov/pubmed/?term=Karpe J[Author]&cauthor=true&cauthor_uid=18205104), [Kajdaniuk D](http://www.ncbi.nlm.nih.gov/pubmed/?term=Kajdaniuk D[Author]&cauthor=true&cauthor_uid=18205104), [Siemińska L](http://www.ncbi.nlm.nih.gov/pubmed/?term=Siemińska L[Author]&cauthor=true&cauthor_uid=18205104), [Głogowska-Szelag J](http://www.ncbi.nlm.nih.gov/pubmed/?term=Głogowska-Szelag J[Author]&cauthor=true&cauthor_uid=18205104), [Foltyn W](http://www.ncbi.nlm.nih.gov/pubmed/?term=Foltyn W[Author]&cauthor=true&cauthor_uid=18205104), [Strzelczyk J](http://www.ncbi.nlm.nih.gov/pubmed/?term=Strzelczyk J[Author]&cauthor=true&cauthor_uid=18205104), [Nowak K](http://www.ncbi.nlm.nih.gov/pubmed/?term=Nowak K[Author]&cauthor=true&cauthor_uid=18205104).

[Endokrynol Pol.](http://www.ncbi.nlm.nih.gov/pubmed/?term=The+blood+concentration+of+intercellular+adhesion+molecule-1+(sICAM-1)+and+vascular+cell+adhesion+molecule-1+(sVCAM-1)+in+patients+with+active+thyroid-associated+orbitopathy+before+and+after+methylprednisolone+treatment.) 2007 Nov-Dec;58(6):487-91.

Reason: 1 no relevant outcomes

13 The effect of long-term prednisone treatment on growth hormone and insulin-like growth factor-1.

[Prummel MF](http://www.ncbi.nlm.nih.gov/pubmed/?term=Prummel MF[Author]&cauthor=true&cauthor_uid=8957747), [Wiersinga WM](http://www.ncbi.nlm.nih.gov/pubmed/?term=Wiersinga WM[Author]&cauthor=true&cauthor_uid=8957747), [Oosting H](http://www.ncbi.nlm.nih.gov/pubmed/?term=Oosting H[Author]&cauthor=true&cauthor_uid=8957747), [Endert E](http://www.ncbi.nlm.nih.gov/pubmed/?term=Endert E[Author]&cauthor=true&cauthor_uid=8957747).

[J Endocrinol Invest.](http://www.ncbi.nlm.nih.gov/pubmed/8957747) 1996 Oct;19(9):620-3.

Reason: no relevant outcomes

14 Methylprednisolone and hepatotoxicity in Graves' ophthalmopathy.

[Wichary H](http://www.ncbi.nlm.nih.gov/pubmed/?term=Wichary H[Author]&cauthor=true&cauthor_uid=22029719), [Gasińska T](http://www.ncbi.nlm.nih.gov/pubmed/?term=Gasińska T[Author]&cauthor=true&cauthor_uid=22029719).

[Thyroid.](http://www.ncbi.nlm.nih.gov/pubmed/?term=Methylprednisolone+and+hepatotoxicity+in Graves' ophthalmopathy.) 2012 Jan;22(1):64-9. doi: 10.1089/thy.2010.0158. Epub 2011 Oct 26.

Reason: no relevant outcomes

15 Effect of prednisone on renal function in man.

[van Acker BA](http://www.ncbi.nlm.nih.gov/pubmed/?term=van Acker BA[Author]&cauthor=true&cauthor_uid=8247189), [Prummel MF](http://www.ncbi.nlm.nih.gov/pubmed/?term=Prummel MF[Author]&cauthor=true&cauthor_uid=8247189), [Weber JA](http://www.ncbi.nlm.nih.gov/pubmed/?term=Weber JA[Author]&cauthor=true&cauthor_uid=8247189), [Wiersinga WM](http://www.ncbi.nlm.nih.gov/pubmed/?term=Wiersinga WM[Author]&cauthor=true&cauthor_uid=8247189), [Arisz L](http://www.ncbi.nlm.nih.gov/pubmed/?term=Arisz L[Author]&cauthor=true&cauthor_uid=8247189).

[Nephron.](http://www.ncbi.nlm.nih.gov/pubmed/8247189) 1993;65(2):254-9.

Reason: no relevant outcomes

16 A pilot study to monitor Graves' ophthalmopathy with a combination of pattern-reversal and motion-onset visual evoked potentials.

[Szanyi J](http://www.ncbi.nlm.nih.gov/pubmed/?term=Szanyi J[Author]&cauthor=true&cauthor_uid=22811284), [Kremlacek J](http://www.ncbi.nlm.nih.gov/pubmed/?term=Kremlacek J[Author]&cauthor=true&cauthor_uid=22811284), [Kubova Z](http://www.ncbi.nlm.nih.gov/pubmed/?term=Kubova Z[Author]&cauthor=true&cauthor_uid=22811284), [Langrova J](http://www.ncbi.nlm.nih.gov/pubmed/?term=Langrova J[Author]&cauthor=true&cauthor_uid=22811284), [Kuba M](http://www.ncbi.nlm.nih.gov/pubmed/?term=Kuba M[Author]&cauthor=true&cauthor_uid=22811284).

[J Clin Apher.](http://www.ncbi.nlm.nih.gov/pubmed/22811284) 2012;27(6):295-301. doi: 10.1002/jca.21243. Epub 2012 Jul 19.

Reason: no relevant outcomes

17 Comparability of proptosis measurements by different techniques.

[Segni M](http://www.ncbi.nlm.nih.gov/pubmed/?term=Segni M[Author]&cauthor=true&cauthor_uid=12036674), [Bartley GB](http://www.ncbi.nlm.nih.gov/pubmed/?term=Bartley GB[Author]&cauthor=true&cauthor_uid=12036674), [Garrity JA](http://www.ncbi.nlm.nih.gov/pubmed/?term=Garrity JA[Author]&cauthor=true&cauthor_uid=12036674), [Bergstralh EJ](http://www.ncbi.nlm.nih.gov/pubmed/?term=Bergstralh EJ[Author]&cauthor=true&cauthor_uid=12036674), [Gorman CA](http://www.ncbi.nlm.nih.gov/pubmed/?term=Gorman CA[Author]&cauthor=true&cauthor_uid=12036674).

[Am J Ophthalmol.](http://www.ncbi.nlm.nih.gov/pubmed/12036674) 2002 Jun;133(6):813-8.

Reason: no relevant outcomes

18 Repeated peribulbar injections of triamcinolone acetonide: a successful and safe treatment for moderate to severe Graves' ophthalmopathy.

[Bordaberry M](http://www.ncbi.nlm.nih.gov/pubmed/?term=Bordaberry M[Author]&cauthor=true&cauthor_uid=18937809), [Marques DL](http://www.ncbi.nlm.nih.gov/pubmed/?term=Marques DL[Author]&cauthor=true&cauthor_uid=18937809), [Pereira-Lima JC](http://www.ncbi.nlm.nih.gov/pubmed/?term=Pereira-Lima JC[Author]&cauthor=true&cauthor_uid=18937809), [Marcon IM](http://www.ncbi.nlm.nih.gov/pubmed/?term=Marcon IM[Author]&cauthor=true&cauthor_uid=18937809), [Schmid H](http://www.ncbi.nlm.nih.gov/pubmed/?term=Schmid H[Author]&cauthor=true&cauthor_uid=18937809).

[Acta Ophthalmol.](http://www.ncbi.nlm.nih.gov/pubmed/18937809) 2009 Feb;87(1):58-64. doi: 10.1111/j.1755-3768.2008.01171.x. Epub 2008 Oct 7.

No relevant outcomes (only CAS)

19 《糖皮质激素联合球后放射治疗Graves眼病》

侯华英;  姜玉华;  程玉峰;  孙嗣伟

《[山东医药](http://lib.cqvip.com/qk/92062X/)》 CAS 2007年第47卷第30期 79-80页,共2页

Unqualified interventions：IVGC+OGC

20 《甲眼消治疗Graves眼病25例临床观察》

廖世煌;  刘清平;  刘丽霞;  李小兵

《[中医杂志](http://lib.cqvip.com/qk/90115X/)》 CSCD 2002年第43卷第8期 606-608页,共3页

Unqualified interventions：traditional Chinese medicine

21 《激素冲击疗法联合局部注射治疗甲状腺相关眼病疗效观察》

[王洁](http://192.168.89.203/cross/search.jsp);  [魏庆芳](http://192.168.89.203/cross/search.jsp)

[中国地方病学杂志](http://192.168.89.203/cross/search.jsp) 2012; 31(2) : 229

Unqualified interventions：ROGC+IV

22 《曲安奈德眶周注射治疗早期甲状腺相关眼病疗效观察》

[乐丽娜](http://192.168.89.203/cross/search.jsp)

[浙江临床医学](http://192.168.89.203/cross/search.jsp) 2011; 13(12) : 1397-1398

Unqualified interventions：only ROGC

23 《大剂量甲基泼尼松龙治疗Graves眼病疗效观察》

江艳 杨慧英 李红 蒋世钊 徐玉善

《海南医学》 2010年第4期,37-39页

Unqualified interventions：IVGC+OGC vs blank control

24 《[激素冲击疗法联合局部注射治疗甲状腺相关眼病疗效观察](http://www.baidu.com/link?url=EQL19HUgZnZN7ICJ-EnkfF0Tf3gwgFkMQDLwDzNqFUriXbVtETUuqOH27dEa66Y_9QaGIoi2bb3mwZEnjaDBca)》

王洁 魏庆芳

[《中华地方病学杂志》](http://xueshu.baidu.com/s?wd=journaluri:(45568b230fba75fc) 《中华地方病学杂志》&tn=SE_baiduxueshu_c1gjeupa&ie=utf-8&sc_f_para=sc_hilight=publish&sort=sc_cited)，2012, 31(2)

1Unqualified interventions：ROGC+IVGC

25 《甲基强的松及球后放疗治疗甲状腺相关眼病》

蒋东艳

实用全科医学 2006年 第4卷 第6期 705页

Unqualified interventions：ROGC+IVGC vs OGC

26《曲安奈德上穹窿结膜下注射治疗甲状腺相关眼病上睑退缩的临床研究 》

[曹雨金](http://192.168.89.203/cross/search.jsp);  [邓爱姣](http://192.168.89.203/cross/search.jsp);  [陈细姝](http://192.168.89.203/cross/search.jsp);  [吴杨](http://192.168.89.203/cross/search.jsp)

[中国卫生产业](http://192.168.89.203/cross/search.jsp) 2013; 10(12) : 68-69

Reason: no relevant outcomes

27 《激素不同途径治疗甲状腺相关眼病的疗效比较》

[罗兴中](http://192.168.89.203/cross/search.jsp);  [赖平红](http://192.168.89.203/cross/search.jsp);  [杨海军](http://192.168.89.203/cross/search.jsp);  [邱莹莹](http://192.168.89.203/cross/search.jsp)

[中国中医眼科杂志](http://192.168.89.203/cross/search.jsp) 2011; 21(6) : 327-329

Reason: no relevant outcomes only CAS
