# Supplementary material for: Common Immunosuppressive Monotherapy for Graves’ Ophthalmopathy: A Meta-Analysis
Source: PLoS One. 2015 Oct 15;10(10):e0139544. doi: 10.1371/journal.pone.0139544 (PMC4607493; doi:10.1371/journal.pone.0139544)
Supplement: S3 Text — (DOC) [file pone.0139544.s003.doc]

**OBJECTIVES**

To assess the effects of Common Immunosuppressive Monotherapies for Graves Ophthalmopathy.

**METHODS**

**Criteria for considering studies for this review**

**Types of studies**

Randomised controlled clinical trials.

**Types of participants**

population: patients had to be diagnosed with active and moderate-to-severe GO.

**Diagnostic criteria**

Our criteria for Graves’ ophthalmopathy are clinical examination in consideration of special symptoms and signs. Additional diagnostic tests such as type-B ultrasonic, CT scan and Thyroid function tests, in which TRAb (TSH receptor antibody)/TBII ( thyrotropin binding inhibiting immunoglobulins) are noted where performed, but not considered essential for study inclusion.

**Types of interventions**

We will consider studies for inclusion where the immunosuppressive therapeutic regimens listed below are compared. 2 of the following were included and used seperately. We will exclude studies where only one trial arm contains a immunosuppressive regimen, or where surgical interventions or placebo are compared.

**Intervention**

a）oral glucocorticoids( OGC)

b）intravenous glucocorticoids ( IVGC)

c）retrobulbar injection of glucocorticoids ( ROGC)

d ) orbital radiotherapy ( OR)

**Types of outcome measures**

**Primary outcomes**

a） Response rate

**Secondary outcomes**

a） Cas reduction

b） Proptosis reduction

**Timing of outcome measurement**

All outcomes must have reported measurements at a minimum of three months follow-up.

**Definition of outcome measurement**

a ) The response rate: the ratio of responders to total number of participants.When the response rate was reported, we used it directly. If not available, a relevant improvement of clinically parameters was identified as the response to therapy: e.g. decrease in proptosis and eyelid retraction of 2 mm or greater, improvement in grade of orbital soft tissue swelling, disappearance of diplopia in primary gaze, and/or improvement of eye movement and visual acuity, respectively.

b ) The standard mean difference in the reduction in clinical activity score(CAS) from baseline to the end of treatment.

c ) The mean difference in proptosis from baseline to the end of treatment.

**Search methods for identification of studies**

We will search th following sources from inception to the present:

• The PubMed.

• EMBASE.

• Cochrane Library databases.

• the Chinese Biomedicine Databas.

**Searching other resources**

We will try to identify other potentially eligible trials by searching the reference lists of retrieved included trials, (systematic) reviews and meta-analyses.

**Data collection and analysis**

**Selection of studies**

Three review authors (P.M, L.H.J, Y.Z) will independently scan the abstract, title, or both sections of every record retrieved, to determine the studies to be assessed further.We will investigate all potentially-relevant articles as full text. We will present an adapted PRISMA (preferred reporting items for systematic reviews and meta-analyses) flow-chart of study selection

**Search Strategy**

All RCTs were identified through a systematic search consisting of (1) an electronic search of PubMed, EMBASE, the Cochrane library and the Chinese Biomedicine Database and (2) manual searches of the reference lists of original reports and review articles that were retrieved via the electronic searches. A broad search strategy combined terms related to Graves’ ophthalmopathy (including a MeSH search using the exploded term ‘Graves’ ophthalmopathy’ and a keyword search using the words ‘thyroid associated ophthalmopathy’ and ‘thyroid eye disease’), terms related to glucocorticoids (including a MeSH search using the exploded term ‘glucocorticoids’ and a keyword search using the words ‘methylprednisolone’ and ‘[prednison](../../../../D:/Program%20Files/Youdao/Dict/6.2.54.2064/resultui/frame/javascript:void(0)%3B)e’), and terms related to orbital radiotherapy (including a MeSH search using the exploded term ‘radiotherapy’ and a keyword search using ‘orbital radiotherapy’). The search was limited to clinical trials.

**Data extraction and management.**

We used a customized form for data extraction. The following data were recorded and extracted: authors, the time of publication, information on study design ( randomization, allocation concealment, intention-to-treat analysis, double or single blind), location of trial, follow-up time, severity, CAS, patient age, sex, race, all outcome measures and other essential information. In addition, we noted the proportion of withdrawals, as well as the number of patients undergoing adverse events.

**Dealing with duplicate publications and companion papers**

we only included the most recent series in case of data collection from the same study population.

**Assessment of risk of bias in included studies**

Three review authors (P.M, L.H.J, Y.Z) will assess the risk of bias of each trial independently. We will resolve possible disagreements by consensus. We will assess the following

criteria:

• Random sequence generation (selection bias).

• Allocation concealment (selection bias).

• Blinding (performance bias and detection bias), separated for blinding of participants and personnel, and blinding of outcome assessment.

• Incomplete outcome data (attrition bias).

• Selective reporting (reporting bias).

• Other bias.

**Measures of treatment effect**

We will express dichotomous data as odds ratios (OR) or risk ratios (RR) with 95% confidence intervals (CI). We will express continuous data as mean differences (MD) with 95% CI.

**Dealing with missing data**

We will obtain relevant missing data from authors, if feasible, and carefully evaluate important numerical data such as screened, randomised patients as well as intention-to-treat (ITT).

**Assessment of heterogeneity**

We will identify heterogeneity by visual inspection of the forest plots and by using a standard Chi^2^ test with a significance level of α= 0.1, in view of the low power of this test. We will examine heterogeneity using the I^2^ statistic, which quantifies inconsistency across studies to assess the impact of heterogeneity on the meta-analysis, where an I^2^ statistic of 75% or more indicates a considerable level of inconsistency.

When we find heterogeneity, we will attempt to determine potential reasons for it by examining individual study and subgroup characteristics.

**Assessment of reporting biases**

We will use funnel plots to assess small study effects.Owing to several possible explanations for funnel plot asymmetry, we will interpret results carefully.

**Data synthesis**

we will perform statistical analyses according to the statistical guidelines contained in the latest version of the Cochrane Handbook for Systematic Reviews of Interventions.
